# Mitochondrial targeting by dichloroacetate improves outcome following hemorrhagic shock

**DOI:** 10.1038/s41598-017-02495-5

**Published:** 2017-06-01

**Authors:** Kumar Subramani, Sumin Lu, Marie Warren, Xiaogang Chu, Haroldo A. Toque, R. William Caldwell, Michael P. Diamond, Raghavan Raju

**Affiliations:** 1 Department of Laboratory Sciences, Augusta University, Augusta, GA 30912 United States of America; 2 Department of Surgery, Augusta University, Augusta, GA 30912 United States of America; 3 Department of Biochemistry and Molecular Biology, Augusta University, Augusta, GA 30912 United States of America; 4 Department of Pharmacology and Toxicology, Augusta University, Augusta, GA 30912 United States of America; 5 Department of Obstetrics and Gynaecology, Augusta University, Augusta, GA 30912 United States of America

## Abstract

Hemorrhagic shock is a leading cause of death in people under the age of 45 and accounts for almost half of trauma-related deaths. In order to develop a treatment strategy based on potentiating mitochondrial function, we investigated the effect of the orphan drug dichloroacetate (DCA) on survival in an animal model of hemorrhagic shock in the absence of fluid resuscitation. Hemorrhagic shock was induced in rats by withdrawing 60% of the blood volume and maintaining a hypotensive state. The studies demonstrated prolonged survival of rats subjected to hemorrhagic injury (HI) when treated with DCA. In separate experiments, using a fluid resuscitation model we studied mitochondrial functional alterations and changes in metabolic networks connected to mitochondria following HI and treatment with DCA. DCA treatment restored cardiac mitochondrial membrane potential and tissue ATP in the rats following HI. Treatment with DCA resulted in normalization of several metabolic and molecular parameters including plasma lactate and p-AMPK/AMPK, as well as Ach-mediated vascular relaxation. In conclusion we demonstrate that DCA can be successfully used in the treatment of hemorrhagic shock in the absence of fluid resuscitation; therefore DCA may be a good candidate in prolonged field care following severe blood loss.

## Introduction

Trauma is the leading cause of death for individuals under the age of 45 years old^1, 2^. Hemorrhage is the most common cause of preventable death in this group^3, 4^. Hypotensive volume replacement permits maintenance of limited tissue reperfusion and continued metabolic activities to maintain cell viability following hemorrhagic shock. However, there is a lack of consensus on the use of any specific resuscitation strategy or adjuncts to fluid resuscitation. Though optimal resuscitation strategy remains controversial, hemorrhage control and resuscitation are high priorities in trauma care in civilian as well as combat situations^5–7^. When administration of fluid resuscitation is logistically difficult, particularly in combat situations, there is a need for agents that prolong survival in the absence of fluid resuscitation in prolonged field care to improve survival.

Severe haemorrhage and shock lead to whole body tissue hypoxia and nutrient deprivation. The severe blood loss leads to prolonged hypotension and impaired coronary flow. This causes decreased cardiac perfusion, myocardial hypoxia and cardiac dysfunction, and exemplify the role of the heart as an important target organ. Hemorrhagic shock is known to cause decreased cardiac output, stroke volume, cardiac contractility, and impaired mitochondrial bioenergetics^8–11^.

Hypoxia and reperfusion injury result in dysregulation of molecular pathways and functions including reduced mitochondrial ATP production^10, 12–15^. These evidences suggest that mitochondria play a critical role in maintaining cellular homeostasis following hemorrhagic shock. Hemorrhagic injury (HI) in animal models have demonstrated decreased activities of electron transport chain complexes, increased release of cytochrome c from the mitochondria, and decreased ATP production^16–19^. Our laboratory and others have demonstrated salutary effect of resveratrol, a mitochondria potentiating agent, following HI in animal models^18, 20, 21^. Treatment with resveratrol and a synthetic sirtuin 1 (SIRT1) activator, SRT1720, prolonged life following HI in our animal model, further indicating the possibility of a critical role for mitochondria in outcome following HI^22^. SIRT1 is a deacetylase that regulates the activity of a number of transcription factors, and modulate mitochondrial biogenesis and function^13, 23^.

We also found increased expression of pyruvate dehydrogenase kinase (Pdk) in the heart of rats subjected to HI and restoration of Pdk expression when the animals were treated with resveratrol following HI^24^. Pdk inhibits pyruvate dehydrogenase (Pdh), a key enzyme that couples glycolytic pathway to the tricarboxylic acid (TCA) cycle by catalysing the decarboxylation of pyruvate to acetyl-CoA^25^. In order to further identify a direct role for mitochondria in HI-mediated organ function and survival, and to test whether inhibition of Pdk can change outcome following HI, we induced HI in rats and treated them with dichloroacetate (DCA) and monitored survival in the absence of fluid resuscitation. DCA is an inhibitor of Pdk and enhances the activity of Pdh, resulting in increased turnover of pyruvate to acetyl CoA thereby augmenting oxidative phosphorylation.

It has been shown that DCA improves cardiac output and left ventricular function in myocardial ischemia^26, 27^, and prevented the transition from cardiac hypertrophy to heart failure in experimental animal models^28^. Furthermore, clinical studies with this small molecule resulted in reduced lactate levels in patients with congenital lactic acidosis and sepsis^29, 30^. The influence of DCA on cellular energetics following hemorrhagic shock in the absence of fluid resuscitation has been unclear. We used our HI models involving no fluid resuscitation as well as the one with fluid resuscitation, to further characterize the influence of DCA on mitochondrial function and survival following HI.

## Results

### DCA improves survival in the absence of fluid resuscitation

**Figure 1:**
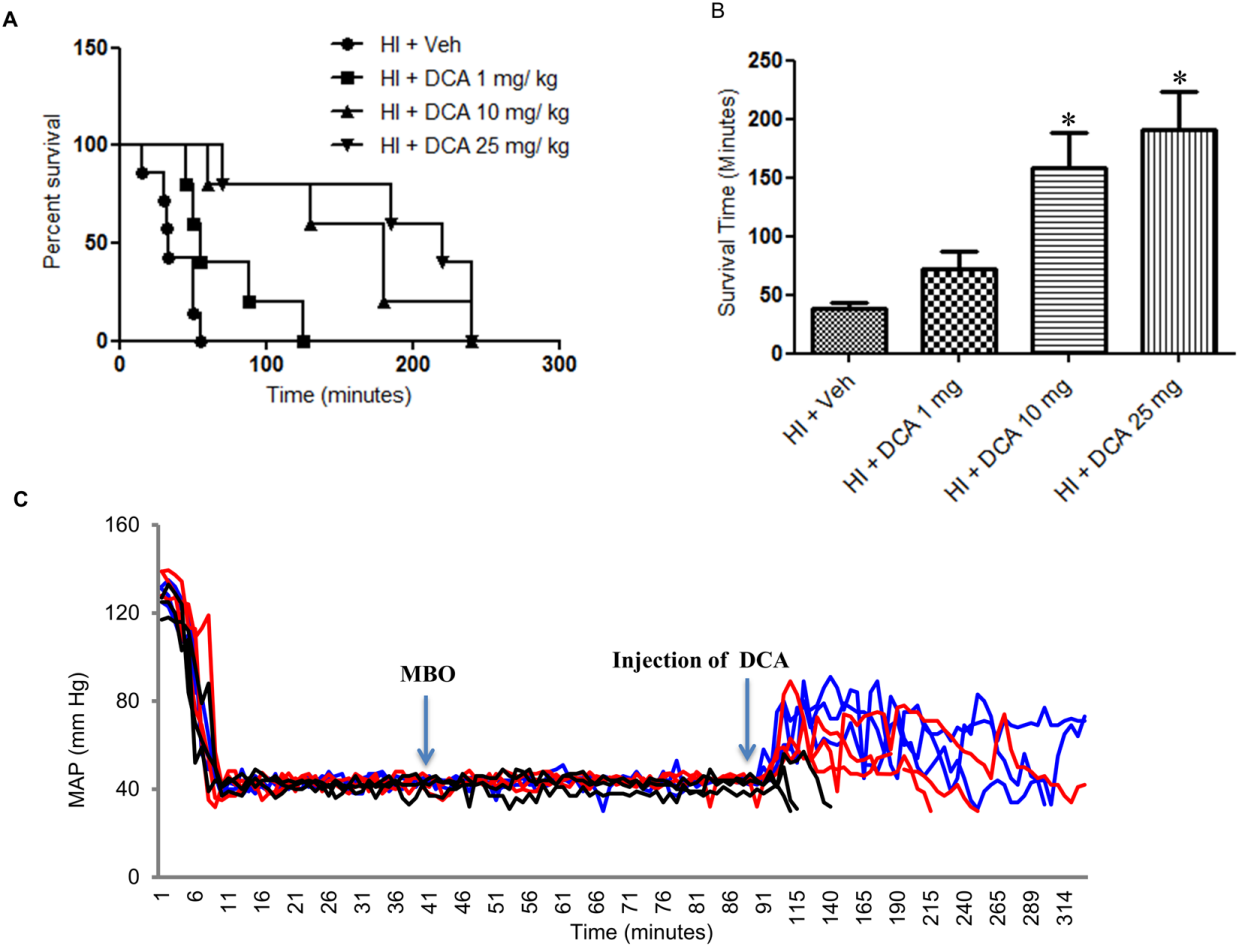
Dichloroacetate (DCA) prolongs life after HI in the absence of resuscitation. (**A)** Kaplan-Meier survival curves. HI + Veh (n = 7), HI + DCA 1 mg/Kg (n = 5), HI + DCA 10 mg/Kg (n = 5) and HI + DCA 25 mg/Kg (n = 5) p < 0.05 versus HI + Veh, all curves. (**B**) Bar diagram represents mean survival time (minutes) in each of experimental groups (mean ± SEM); *indicates p < 0.05 compared HI + Veh; (**C)** Time course of mean arterial pressure (MAP) from the onset of hemorrhage to immediately prior to euthanasia in representative animals in HI + Veh (black lines), HI + DCA 10 mg/Kg (red line) and HI + DCA 25 mg/Kg (blue line); only three representative animals shown for each group. MBO = maximum bleed out, time at which hemorrhage ended.

DCA is known to potentiate mitochondrial function. Therefore we first tested whether treatment with DCA can prolong survival following HI. In the experimental model of HI we subjected rats to soft tissue trauma and severe hemorrhage, but were not resuscitated with fluid following hypovolemic shock period. Immediately following the shock period, an intravenous dose of DCA was given in 500–600 μl Ringers lactate. The control animals received only 500–600 μl Ringers lactate. We tested three different doses of DCA, 1 mg, 10 mg and 25 mg per Kg body weight and found that the latter two doses significantly improved survival following HI (Fig. 1A,B and C). However, though 25 mg/Kg dose had a slight survival advantage, it was not significant from that achieved by 10 mg/Kg dose (Fig. 1A). The mean survival times for each these doses, including vehicle were 38 min, 73 min, 160 min and 200 min for 0, 1, 10 and 25 mg/Kg doses respectively (Fig. 1B). None of the animals that did not DCA (vehicle only) survived more than 55 minutes and the survival period ranged from15–55 minutes. It is important to note that unlike the control animals, none of the animals that received 10 or 25 mg/Kg dose of DCA died within the first 60 minutes. From Fig. 1C, it is also evident that DCA administration resulted in elevated mean arterial pressure (MAP).

### Effect of DCA on hemodynamics after fluid resuscitation

**Figure 2:**
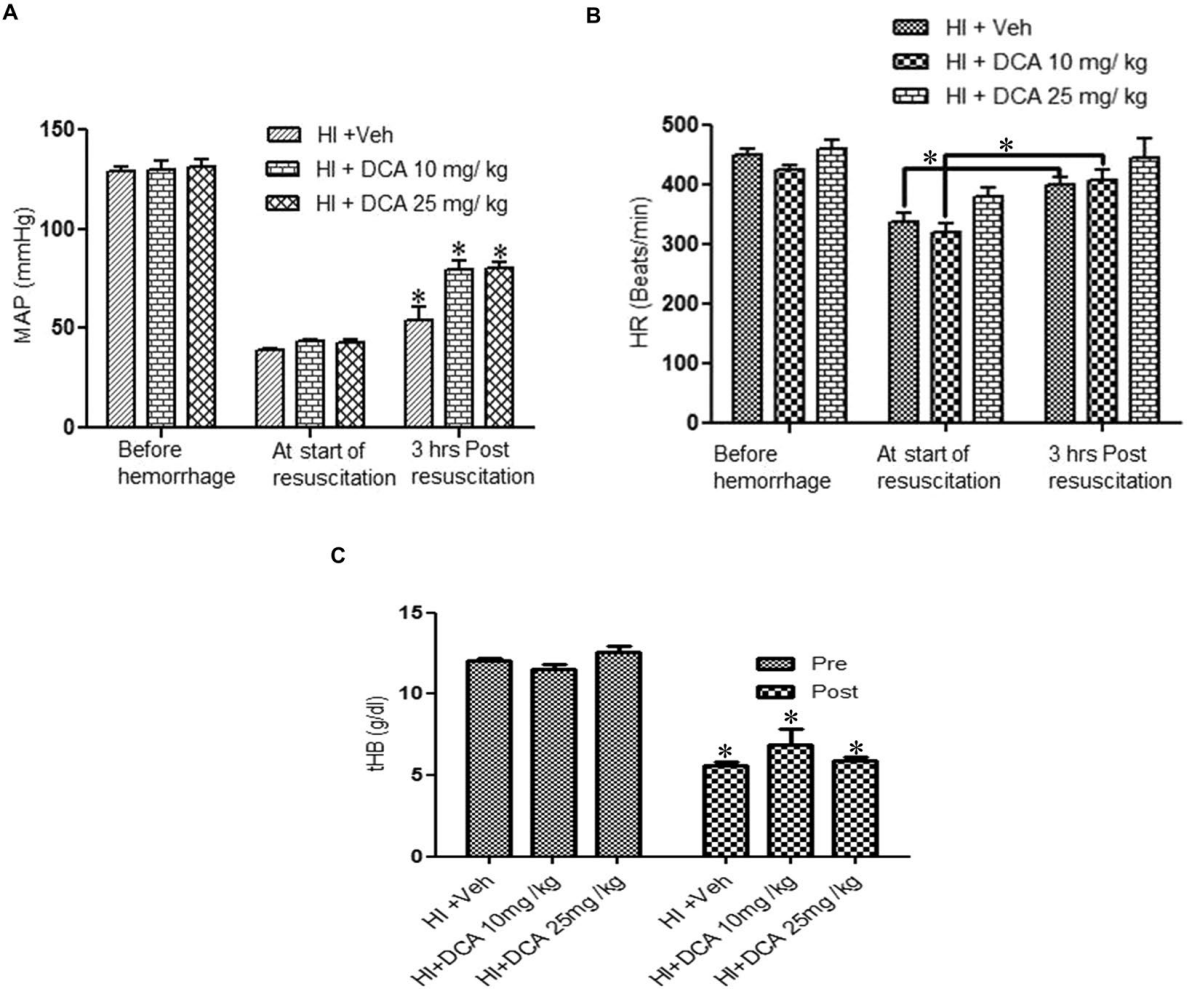
Hemodynamics following HI. (**A**) Mean arterial pressure (MAP). MAP before hemorrhage, at the start of fluid resuscitation and 3 hours post resuscitation. (**B**) Heart rate (HR). HR before hemorrhage, at the start of fluid resuscitation and 3 hours post resuscitation; groups. For panels A and B: HI + Veh (n = 8), HI + DCA 10 mg/Kg (n = 6) and HI + DCA 25 mg/Kg (n = 6). *p < 0.05 for respective groups compared to the MAP at the start of fluid resuscitation. (**C**) Blood Hemoglobin (tHB) levels before and at 3 hours post resuscitation in HI + Veh, HI + DCA 10 mg and 25 mg; n = 6. *Indicates p < 0.05 compared to pre-hemorrhage.

**Figure 3:**
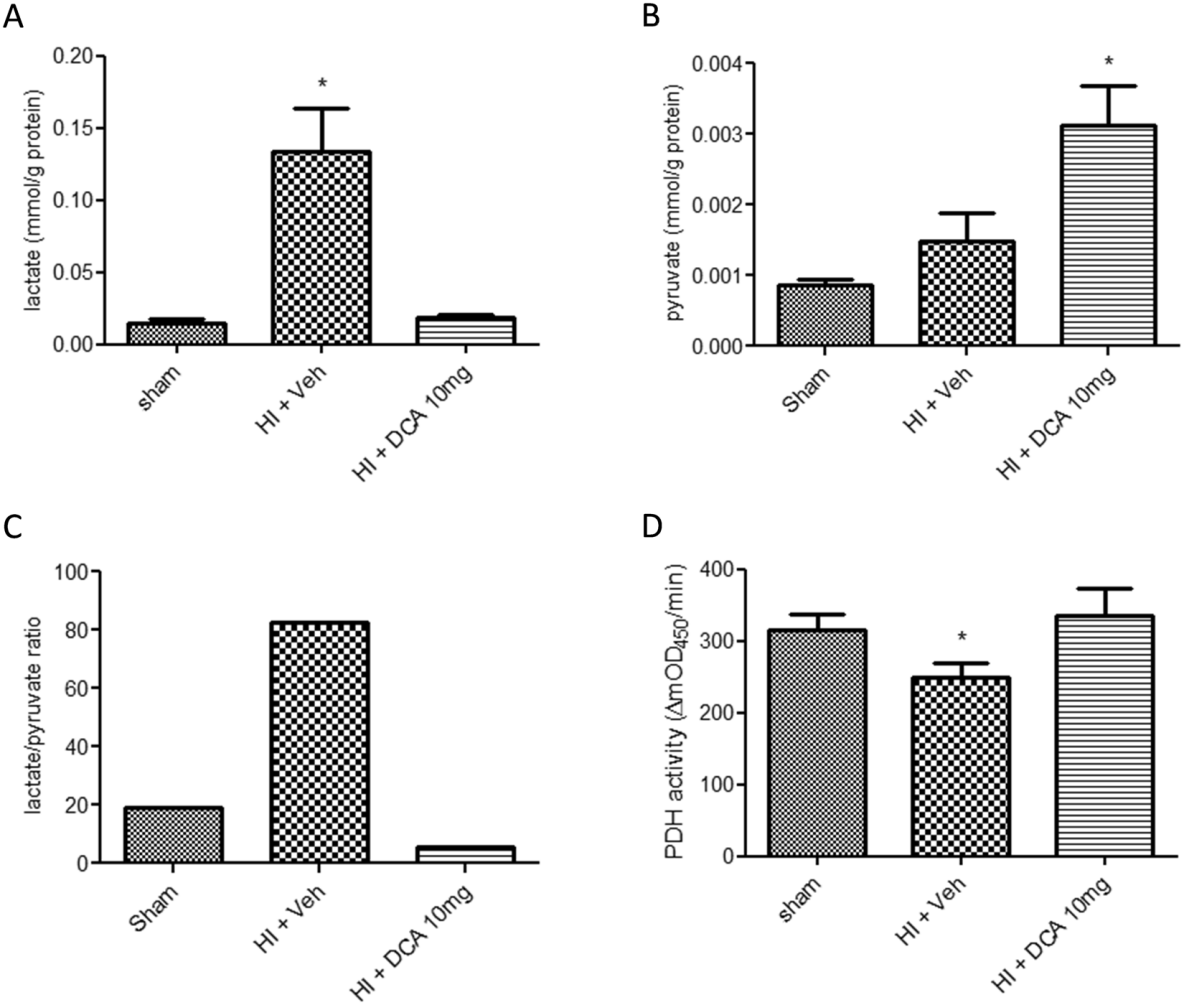
Plasma lactate, pyruvate and myocardial PDH. Plasma lactate (**A**) and plasma pyruvate (**B**) were measured as per manufacturers’ protocol. Results were normalized to plasma proteins to account for hemodilution. Lactate/pyruvate ratio (**C**) was calculated from the mean values. PDH activity (**D**) was measured in the heart tissue using 200 ug of total protein. All samples were tested in duplicates. Groups: Sham, HI + Veh, HI + DCA 10 mg. Bars indicate mean ± SEM. *Indicates p < 0.05 vs Sham.

In the next experiment, the effect of DCA on hemodynamics and molecular parameters following HI was tested using the fluid resuscitation model. HI was induced and fluid resuscitation was carried out with Ringers Lactate, two times the volume of shed blood, for one hour. DCA or vehicle was administered 10 minutes after the start of fluid resuscitation. MAP and heart rate were observed for 3 hours and the animals were then sacrificed. Blood gas values were measured before hemorrhage and after the end of the study (Table 1). As shown in Fig. 2A, following fluid resuscitation MAP increased significantly in all three groups with significant increase of MAP in the DCA-treated groups (10 mg and 25 mg) compared to untreated groups. Nevertheless, none of the resuscitation groups fully achieved restoration of MAP equal to the MAP prior to haemorrhage. There was also an increase in the heart rate in all the three groups of animals following fluid resuscitation; the difference was significant in both vehicle group and the group of rats that received 10 mg/Kg DCA (Fig. 2B) when compared to respective groups at the start of fluid resuscitation. A significant and comparable blood loss was evident in all the three groups (Fig. 2C). As it is apparent, the decrease in total haemoglobin was consistent with the severe blood loss. The difference in plasma lactate was very pronounced at 3 hours following fluid resuscitation (Fig. 3A) in the vehicle treated rats subjected to HI. The lactate levels were almost normalized in the rats treated with DCA. The lactate/pyruvate ratio also improved significantly following DCA treatment (Fig. 3A–C). This was followed by an increased activity of myocardial PDH (Fig. 3D).

**Table 1:** Blood gas and hematocrit changes with HI and DCA treatment.

Group	Pre (n = 15)	HI + Veh (n = 5)	HI + DCA 10 mg (n = 5)	HI + DCA 25 mg (n = 5)
_P_H	7.40 ± 0.02	7.27 ± 0.62*	7.39 ± 0.04	7.38 ± 0.05
_P_CO_2_(mmHg)	36.72 ± 1.76	24.88 ± 3.35*	32.78 ± 1.61	30.16 ± 2.04
_P_O_2_(mmHg)	116.28 ± 3.96	142.86 ± 17.85	138.66 ± 5.19	115.03 ± 22.11
tCO_2_(mmol/L)	23.25 ± 0.81	13.78 ± 2.34*	20.92 ± 1.86	17.76 ± 3.84
HCO_3_(mmol/L)	22.13 ± 0.57	13.02 ± 2.28*	19.94 ± 1.84	16.9 ± 3.73
stHCO_3_(mmol/L)	22.54 ± 0.64	15.14 ± 2.22*	21.02 ± 1.89	18.96 ± 3.08
HCt (%)	36.76 ± 0.64	17.52 ± 1.29*	20.73 ± 2.73*	17.76 ± 0.48*

Arterial blood was sampled before hemorrhage (Pre) and at 3 hours post resuscitation. *p < 0.05. All the parameters were measured using a blood gas analyzer using 120 ul of blood. Abbreviations: pCO_2_ = partial pressure of CO_2_; pO_2_ = partial pressure of oxygen; tCO_2_ = total carbon dioxide; HCO_3_− = bicarbonate; stHCO_3_− = standard bicarbonate; HCt = hematocrit.

### DCA targets mitochondria

**Figure 4:**
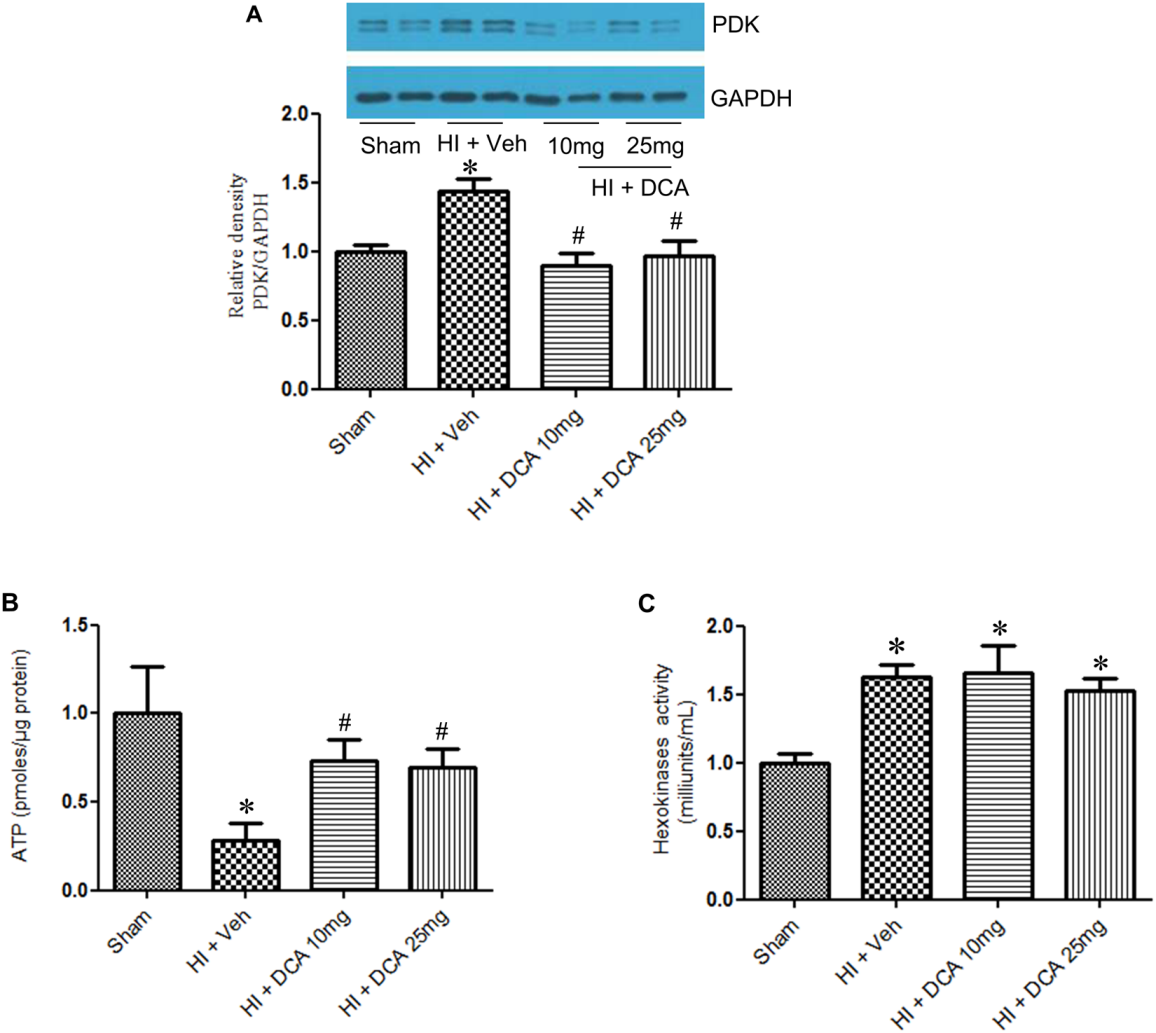
Pdk expression, ATP and hexokinase activity following HI and DCA treatment (Heart). (**A**) Pdk expression in Sham, HI + Veh, HI + DCA 10 mg and HI + DCA 25 mg. The Western blot images are representatives of three independent experiments, scanned from the original and without alteration. (**B**) ATP in heart tissues following HI and DCA treatment. ATP level was measured using a kit from Life Technologies - Molecular Probes. (**C**) Hexokinase activity following HI and DCA treatment: Hexokinase activity was measured by colorimetric method as described in methods section. Groups: Sham, HI + Veh, HI + DCA 10 mg and 25 mg. Bars indicate mean ± SEM; (n = 6), *indicates p < 0.05 for Sham vs respective groups.

DCA is well described as an inhibitor of the mitochondrial enzyme pyruvate dehydrogenase kinase (Pdk)^31, 32^. In order to test the effect of DCA treatment on Pdk, we performed Western blot on the heart tissue isolated from rats treated with vehicle or DCA following HI. As shown in Fig. 4A, we observed a significant increase in Pdk expression in the heart following HI. As Pdk inhibits the enzyme pyruvate dehydrogenase (PDH) that catalyses the conversion of pyruvate to acetyl CoA, the increased Pdk expression following HI was concomitant with a decreased level of ATP in the heart (Fig. 4B). The protein expression levels of Pdk were restored to normal levels in rats that received DCA at the dose of 10 and 25 mg/Kg. We observed a corresponding increase in total ATP in the heart tissue demonstrating increased mitochondrial respiratory output. As severe haemorrhage leads to hypoxia, the elevation of Pdk levels and diminished ATP levels indicated impaired mitochondrial function. In order to determine whether a mitochondrial glycolytic shift takes place following HI, and if a reversal follows DCA treatment, we tested the activity of the glycolytic enzyme hexokinase and observed significant increase (Fig. 4C). However, we did not see a decline in the hexokinase activity following DCA treatment indicating a maintenance of heightened enzyme activity even after mitochondrial ATP output increased. Hexokinase may be too far upstream to identify a DCA-mediated effect. It may also be an adaptive mechanism to increase pyruvate generation to support TCA cycle.

### Molecular mediators in DCA mediated salutary effect

**Figure 5:**
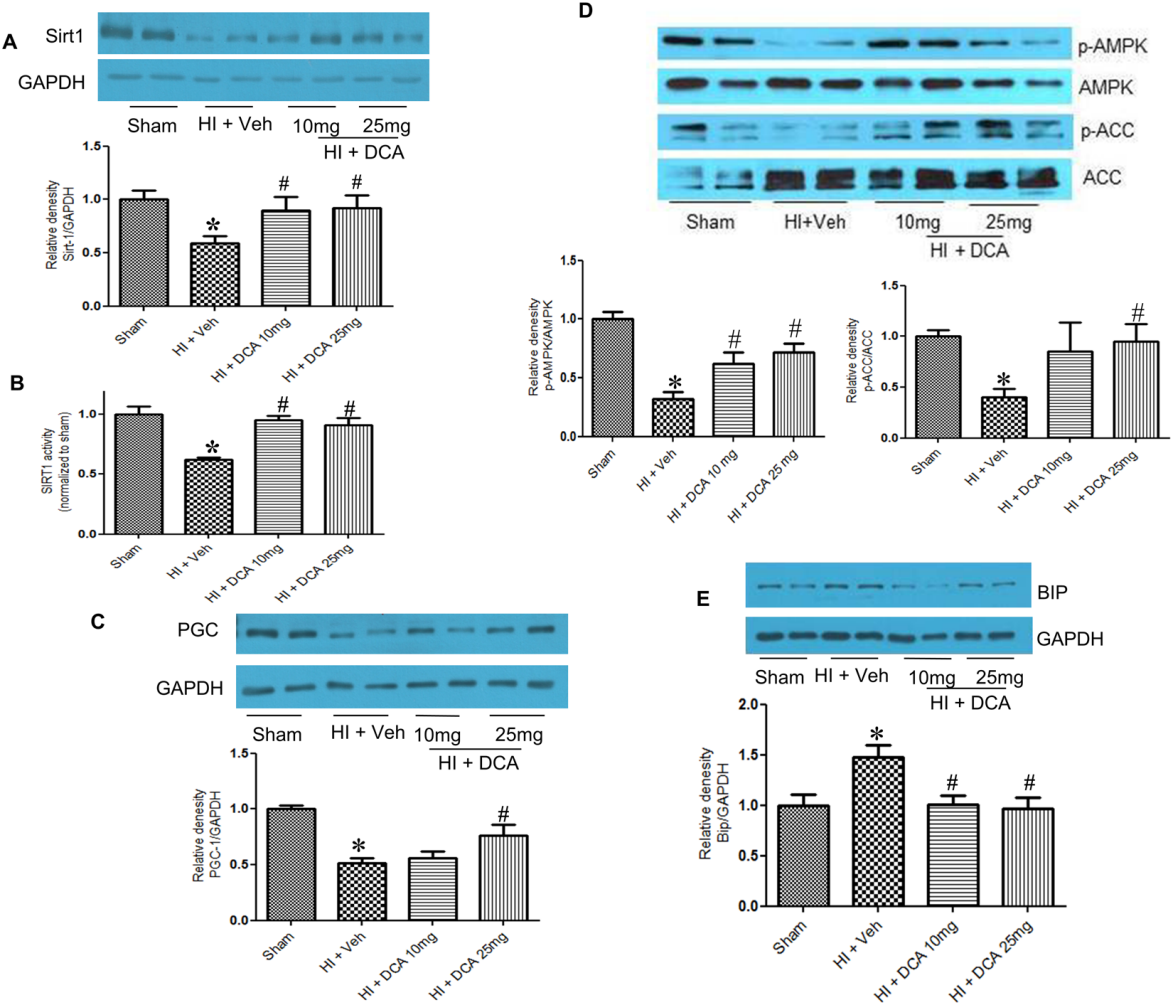
Molecular mediators of mitochondrial function. (**A**) Sirt1 expression following HI and DCA treatment. (**B**) Sirt-1 enzyme activity, results were normalized to sham levels. (**C**) Pgc-1α expression following HI and DCA treatment. (**D**) p-AMPK/AMPK, p-ACC/ACC expression following HI and DCA. (**E**) BIP expression following HI and DCA treatment. The Western blot images are representatives of 3-4 independent experiments. Bars indicate mean ± SEM; (n = 6); ^#^indicates p < 0.05 for HI + Veh vs respective groups.

In order to identify the molecular mediators involved in the DCA-induced salutary effect following HI we performed a series of experiments to test changes in the levels of key proteins related to mitochondrial function. Based upon our previous studies that showed significant impairment of cardiac function following HI, we focussed our molecular studies on heart tissue. One of the critical proteins involved in the post-translational activation of transcription factors related to mitochondrial function is SIRT1. The amount of this deacetylase enzyme was significantly decreased in the heart of rats following HI and fluid resuscitation (Fig. 5A). In the animals that received DCA at 10 mg/Kg and 25 mg/Kg dose, the protein level was similar to that was observed in sham operated animals (Fig. 5A). Consistent with the change in expression level of SIRT1 following HI and DCA treatments, we also observed a significant decrease in the activity of SIRT1 in rats that underwent HI and restoration of the activity in DCA treated rats (Fig. 5B). Both 10 mg/Kg and 25 mg/Kg DCA doses maintained SIRT1 activity similar to that in the sham. Among the major targets of SIRT1 is Pgc-1α, a mitochondrial biogenesis factor. The transcriptional cofactor Pgc-1α was significantly reduced in the heart of rats subjected to HI (Fig. 5C). Though the protein expression was not significantly increased with the 10 mg dose of DCA, there was a significant restoration of expression in rats that received 25 mg/kg dose. The two proteins that are important in the activation of Pgc-1α are SIRT1 and AMPK. AMPK and p-AMPK levels were determined by Western blot and we observed a significant decrease in the phosphorylated fraction of AMPK following HI (Fig. 5D). However the levels were significantly increased in DCA treated rats when compared to rats that were given only vehicle following HI. Acetyl Co-A carboxylase (ACC) is a direct target of activated AMPK. Therefore, to further confirm the activation of AMPK, we tested whether ACC phosphorylation is altered when rats were subjected to HI and fluid resuscitation with or without DCA treatment. We found the pattern of phosphorylation to be similar to that of AMPK, with significant decrease in HI group and normalization of phosphorylation levels in DCA treated groups, both 10 and 25 mg/kg doses (Fig. 5D) establishing activation of AMPK with DCA treatment. We finally tested one of the markers of endoplasmic reticulum (ER) stress, the ER chaperone Bip, and found its expression significantly elevated following HI, and normalized when treated with DCA (Fig. 5E).

### Mitochondrial membrane potential following HI and DCA treatment

**Figure 6:**
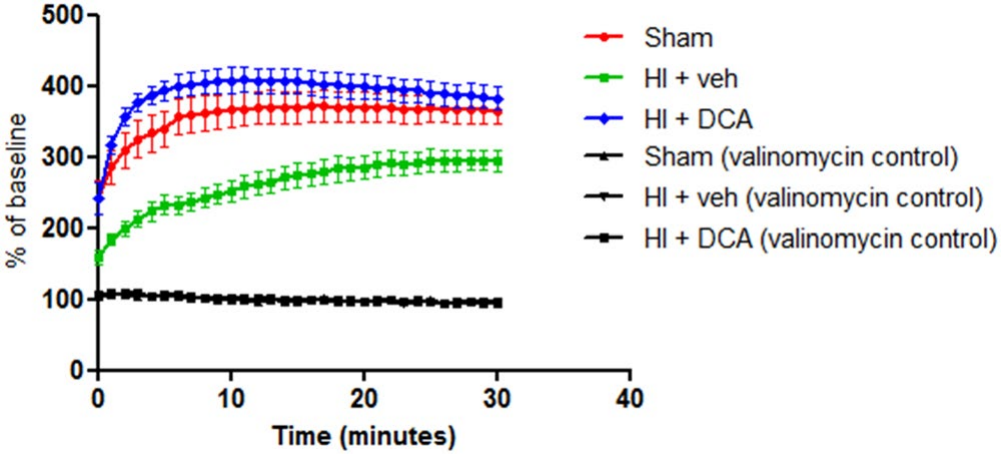
Mitochondrial potential following HI and DCA treatment. JC1 Staining Assay carried out using isolated mitochondria as per the protocol (Sigma, MO). Valinomycin used as an internal control. All samples were tested in triplicates. *Indicates p < 0.05 for HI + Veh compared to Sham. (n = 3–6).

In order to test whether there is change in mitochondrial membrane potential following HI, we performed the JC1 dye uptake assay using mitochondria isolated from the heart^33^. There was a significant decrease in the ratio of aggregate/monomer of the dye as measured by fluorescence intensity ratio at 590/485 in the mitochondria isolated from the heart of rats subjected to HI and fluid resuscitation (Fig. 6). In cells with decreased mitochondrial membrane potential, the aggregates of dye decreases and emit green fluorescence. The fluorescence intensity ratio was not significantly different in the mitochondria isolated from rats treated with DCA compared to that from the sham rats.

### Vascular function in HI and following DCA treatment

**Figure 7:**
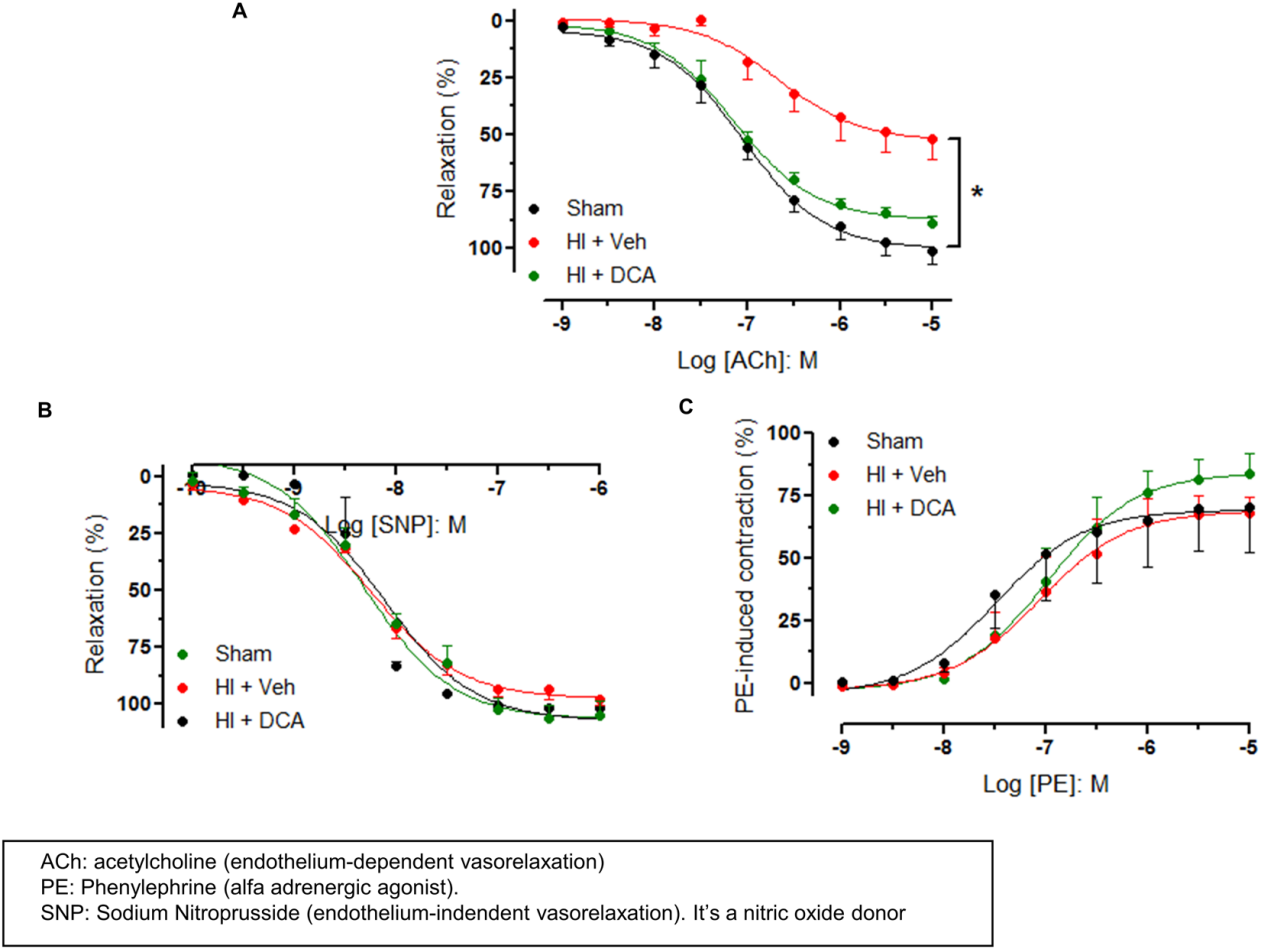
Impairment of endothelium-dependent vasorelaxation in rat aortas. (**A**) Concentration-response curves to ACh (0.001–10 µM), (**B**) sodium nitroprusside (SNP; 0.0001–10 µM), (**C**) and phenylephrine (PE, 0.001–10 µM). Data represent the means ± SEM. n = 5 experiments. *p < 0.05 compared to control group.

To understand the mechanism of salutary effect of DCA we tested the vascular function in aortic rings isolated from the rats subjected to HI, HI with DCA treatment, and sham operation. In this experiment we specifically determined changes in vascular relaxation following HI and DCA treatment. We compared the vascular relaxation response to acetylcholine (Ach), and sodium nitroprusside (SNP), and contractile responses to phenylephrine (PE), using isolated aortic rings from sham operated rats and vehicle-treated or DCA-treated rats following HI. When the aortas were treated with increasing concentrations of Ach or SNP, progressive relaxation was observed with Ach or SNP in all the three groups (Fig. 7A and B). Similarly progressive contraction was seen when treated with PE (Fig. 7C). The aortic rings from the sham and DCA treated animals demonstrated comparable vascular endothelial-dependent relaxation to Ach as demonstrated by similar robust responses, as opposed to impaired responses observed with animals subjected to HI and treated with vehicle^34^ (Fig. 7A). The relaxation responses to the NO donor sodium nitroprusside (SNP), an endothelium-independent relaxation agent, were not different among the groups. Contractile responses to PE also were not different among the groups (Fig. 7B and C).

## Discussion

Hemorrhagic shock and fluid resuscitation cause global ischemia-reoxygenation injury and may lead to organ dysfunction and death. The advances in intensive care and patient management have improved short-term survival following severe injury^35, 36^ however, the overall hospital mortality for trauma patients admitted to the intensive care unit remains high^15, 37^. In combat and civilian situations where care is delayed or fluid resuscitation is logistically difficult, methods to prolong life in the absence of fluid resuscitation are necessitated. Therefore, far-forward management of severe hemorrhage in combat remains a challenge. Though the precise mechanisms of tissue injury and organ dysfunction following hemorrhagic shock are unclear, recent studies support the hypothesis that cellular energetics play a critical role in outcome following hemorrhagic shock^17, 38–40^. Therefore, achieving a protracted period of cellular homeostasis following hemorrhagic shock by treatment with mitochondria potentiating agents is clinically important.

Several studies have demonstrated a decline in mitochondrial function in HI^41, 42^. Our recent studies have shown that agents such as resveratrol and SRT1720 that have profound effects on mitochondria can rescue rats following HI in the absence of fluid resuscitation^22^. Resveratrol was previously shown to improve heart, kidney and liver function after HI in the rodents by several laboratories including ours^20, 43, 44^. Though improved mitochondrial function has been observed, there was a lack of direct evidence establishing the role of mitochondria in HI or recovery. The whole body hypoxia and nutrient deprivation following HI and reoxygenation injury following fluid resuscitation further complicates the molecular signature of HI. A mitochondrial-glycolytic shift due to hypoxic conditions prevailing after severe hemorrhage has been previously proposed^24^.

Based upon the above premise we performed a systematic investigation of the effect of DCA on survival and mitochondrial function following HI. As shown in Fig. 1, DCA significantly improved survival in the absence of fluid resuscitation. We tested three different doses of DCA and two higher doses, 10 mg/Kg and 25 mg/Kg, were most effective in improving survival. This experiment demonstrate that in the absence of fluid resuscitation, an appropriate dose of DCA can prolong life for 2–3 hours following severe hemorrhage and shock in the rats, though some of the rats survived for the entire duration of the observation (4 hours). The mechanism of action of DCA is well studied and it is attributed to inhibition of Pdk^31, 45, 46^. During hypoxic conditions, the mitochondrial gatekeeper Pdh is inhibited by Pdk preventing conversion of pyruvate to acetyl CoA. Under hypoxic conditions cells limit oxidative phosphorylation due to decreased availability of electron acceptors thereby decreasing mitochondrial oxidation. Inhibition of pyruvate decarboxylation to form acetyl CoA is a critical step in this process. The scenario in cellular response to injury is very similar to the Warburg effect which postulated glucose metabolism yielding lactate in cancer cells despite the availability of oxygen, which is distinct from that of normal cells^47^. Nevertheless a profound inhibition of Pdh by Pdk is observed in glycolytic activity within cancer cells and inhibition of Pdk by DCA has been found to improve mitochondrial oxidative phosphorylation^48^. DCA can also significantly induce apoptosis of epithelial ovarian cancer cells^49^. Our experiments demonstrated an increased expression of Pdk in rats subjected to HI and restoration to normal levels following DCA administration. Warburg suggested a mitochondrial- glycolytic shift that results in production of ATPs by glycolysis^50^. A very similar situation may be envisaged in HI with a declining mitochondrial function and an active glycolytic process as evidenced by increased hexokinase activity and decreased total ATP. Though we expected a normalized hexokinase activity in DCA treated group, we did not observe a decrease in its activity from that of vehicle treated rats subjected to HI. This demonstrates that the heightened glycolytic activity continues even after DCA treatment.

The JC-1 uptake assay demonstrated decreased membrane potential in the animals treated only with vehicle. The membrane potential in DCA treated animals, as measured by JC-1 aggregation, was similar to that in sham animals. The experiment demonstrates an overall decline in membrane potential following HI and provides further evidence for the deleterious effect of HI on mitochondria and the salutary actions of DCA in potentiating mitochondrial function. These experiments demonstrate that by directly intervening on mitochondria, the outcome following HI can be significantly improved. The decompensated hemorrhagic shock model we used is well-validated, and a depressed cardiac function and myocardial impairment have been well documented in this model^16, 20^. When DCA is administered intravenously following HI, it is unclear whether the effect of DCA is predominantly in any particular tissue including the heart. Nevertheless the experiments demonstrate a salutary effect in the heart at the cellular level following DCA treatment after HI.

The experiment to test vascular function demonstrated impaired endothelial-dependent vasorelaxation in response to Ach in rats following HI, when compared to DCA-treated rats or sham operated rats. We did not observe any significant differences among the groups in response to phenylephrine or sodium nitroprusside indicating that vascular dysfunction following HI is endothelium-dependent. The involvement of nitric oxide in HI-induced vascular dysfunction and the effect of DCA needs to be further examined, nevertheless the observation is consistent with previous findings of depressed endothelial function following HI^51, 52^.

We observed a significant decrease in the activity of SIRT1 following HI and the levels were normalized with DCA treatment. The effect of DCA treatment on SIRT1 is likely secondary due to improved mitochondrial function, though SIRT1 is known to have profound influence on mitochondrial function. SIRT1 deacetylates Pgc-1α which is a mitochondrial biogenesis factor, and a cofactor for Ppar-α. However the necessary and sufficient conditions for the activation of Pgc-1α are its deacetylation and phosphorylation. Whereas deacetylation is carried out by SIRT1, AMPK plays a major role in the phosphorylation of Pgc-1α. Our experiments demonstrated increased ratio of p-AMPK/AMPK in the heart of rats treated with DCA and subjected to HI. The functional relevance of the activated AMPK is further confirmed by increased phosphorylation of acetyl CoA carboxylase (ACC). ACC is a direct phosphorylation target of AMPK^53^. The results are consistent with a previous report that showed increased activation of AMPK when treated with DCA^54^. The AMPK activation was reported to be due to increased OXPHOS activity. When the animals are treated with DCA, following HI, there is a switch to pyruvate from lactate and the increased OXPHOS activity is expected to consume NADH thereby increasing the NAD^+^/NADH ratio resulting in increased SIRT1 activity. Nevertheless, there is a decrease in SIRT1 activity following HI that is restored with DCA treatment. An inter-relationship between sirtuin activity and AMPK has been proposed elsewhere^55^ suggesting that AMPK activation may also be regulated by sirtuin activity. It is known that activation of SIRT1 leads to deacetylation and activation of the AMPK kinase LKB1, resulting in AMPK activation^56–59^. Furthermore potentiating SIRT1 activity by resveratrol was also followed by increased ATP and AMPK activation^60^.

DCA has been used to treat metabolic disorders such as lactic acidosis and diabetes mellitus due to its ability to reduce circulating glucose and lactate^61^. DCA treatment was shown to maintain normal circulating lactate levels in patients with congenital lactic acidosis^30^. DCA has also been tested in various cancers and other disorders characterized by hypoxia or mitochondrial dysfunction, but with side effects such as peripheral neuropathy due to chronic use^30, 62, 63^. The proposed use of DCA as described in this study does not require chronic use. Therefore side-effects or efficacy issues due to chronic use become less relevant in a single use study for acute conditions such as trauma. There have also been previous attempts testing the effect of DCA in hemorrhagic shock using experimental animal models, but with varying results^64, 65^. However, these studies utilized a very high concentration of DCA (150 mg/Kg) and a constant pressure model with unknown or varying shed blood volumes. The dose response study in our model shows that 10–25 mg/Kg body weight elicits a maximum survival response. Furthermore, the data presented in this study demonstrate that the survival benefit due to DCA treatment is better than that due to resveratrol treatment reported in a previous study^22^. The previous study using resveratrol at the maximum employed dose of 10 mg/Kg demonstrated an average survival period of ~110 minutes with at least one of the six animals having survived the full duration of the study (4 hrs)^22^. However in the current study, the maximum mean survival time following DCA treatment was almost doubled (~200 minutes) and most of the animals survived longer, demonstrating that DCA has a more potent effect. A combinatorial therapy with DCA and resveratrol may also be pursued.

Our results show that the concerted mechanisms initiated following potentiation of mitochondrial function triggers a network of cellular responses to allow a protracted period of metabolic homeostasis. This is further substantiated by the observation of a decrease in the endoplasmic reticulum (ER) chaperone protein Bip with HI. HI is characterized by whole body hypoxia and nutrient deprivation. One of the first responders to hypoxia and nutrient deprivation is the ER which signals and readies the cells by an unfolded protein response or ER stress^66, 67^. These results suggest that cells regain homeostatic balance after HI when treated with DCA; this could be a reason for increased survival observed in the rats even in the absence of fluid resuscitation.

Collectively, the interrogation of metabolic checkpoints following HI demonstrates a major shift in metabolic coordinates with DCA treatment resulting in improved mitochondrial function and faster restoration of homeostatic balance. We therefore conclude that agents that potentiate mitochondrial function will be useful in the treatment of hemorrhagic shock.

## Methods

### Animals

Male Sprague Dawley rats 295 ± 10 g were obtained from Charles River Laboratory (Wilmington, MA, USA). All animal experiments performed in this study were approved by the Institutional Animal Care and Use Committee (IACUC) at Augusta University. All methods were performed in accordance with the relevant guidelines and regulations. All animals were housed in Augusta University animal facility during the experiments as per the approved protocol.

### Hemorrhage injury procedure

Rats were subjected to sham or hemorrhagic injury procedure as described before^22^. Briefly, following the hemorrhage and shock periods rats were subjected to one of the following two procedures: Procedure 1 (called non-resuscitation model) involved no fluid resuscitation, but the survival was monitored for a total of 4 hours. Procedure 2 (called resuscitation model) involved fluid resuscitation with 2X Ringer’s lactate and euthanasia after three hours. Hemorrhage and Shock Method: The animals, fasted overnight, were anesthetized with 2.5% isoflurane (Henry Schein, Dublin, OH, USA) and a midline laparotomy (5 cm) was performed, the incision was closed aseptically in two layers with sutures (Ethilon 5/0, Ethicon, AD Surgical, Sunnyvale, CA, USA). Two femoral arteries and one femoral vein were cannulated (PE-50 tubing), one artery was connected to a blood pressure analyzer (Digi-Med; Micro-Med Inc., Louisville, KY, USA) to monitor mean arterial pressure (MAP); bleeding was performed through the other artery. The resuscitation fluid and agents were administered through the femoral vein. Surgical sites were bathed with bupivacaine. Sham animals were not subjected to bleeding or fluid resuscitation, but they were subjected to laparotomy and groin incisions. Upon awakening, the animals in the HI groups were bled rapidly to a MAP of 40 ± 5 mmHg. The bleeding was continued for 45 min, maintaining the low MAP, until 60% of circulatory blood volume was withdrawn. Following the maximum bleed out (MBO) the animals were maintained in the state of shock by maintaining MAP at 40 ± 5 for another 45 min. Following this, either of the two procedures mentioned below was performed depending on the experimental goal. Blood gas values were measured using Opti-Medical (Roswell, GA) OPTI CCA-TS2 analyzer.

### Non-Resuscitation Model

The animals subjected to this procedure did not receive resuscitation fluid following the above shock period. The experimental animals were randomly divided into four groups. One of the three doses of dichloroacetate (DCA, Sigma Aldrich, St. Lois, MO) was administered intravenously at the end of the shock period to the respective groups. In the last group only vehicle was administered. 500–600 μl of Ringer’s Lactate (RL) was used as vehicle. The animals were continuously observed for 4 hrs and as death cannot be an endpoint as per the IACUC policy, animals were euthanized when their MAP dipped below 30 mmHg.

### Resuscitation model

The animals that underwent this procedure were subjected to fluid resuscitation by administering Ringers lactate, two times the volume of shed blood. Fluid resuscitation was performed for 1 hour from the end of shock period. DCA or vehicle was administered 10 min following the start of fluid resuscitation. After fluid resuscitation, the animals were observed for three hours following which they were euthanized, plasma collected and tissues were harvested for molecular studies.

### Lactate assay

Heparinized blood samples were obtained prior to euthanasia. The plasma was separated by centrifugation (2,000 g, 10 min) and stored at −80 °C until assayed for lactate levels. An aliquot of plasma was used to determine protein concentration (Bio-Rad DC Protein Assay). Lactate levels were measured using a Lactate Assay Kit (Sigma St. Louis, MO 63103 USA) and expressed in relation to the total protein to account for hemodilution.

### Pyruvate assay

Pyruvate levels were measured using a fluorometric Pyruvate Assay Kit (Cayman Chemical Company, Ann Arbor, Michigan) according to the manufacturer’s directions and normalized to plasma total protein (Bio-Rad DC Protein Assay).

### PDH assay

Heart tissues were prepared and PDH enzyme activity levels were measured at 450 nm using a colorimetric PDH Enzyme Activity Kit (Abcam, Cambridge, MA) according to the manufacturer’s directions. Briefly, The PDH enzyme is immunocaptured within the wells of the microplate included with the kit. Activity is determined by following the reduction of NAD+ to NADH, coupled to the reduction of a reporter dye to yield a coloured reaction product with an increase in absorbance at 450 nm at room temperature.

### SIRT1 activity assay

The enzymatic activity of Sirt1 in the heart tissue was assayed by a fluorimetric assay using the SensoLyte Green Sirt1 assay kit (AnaSpec, Fremont, CA, USA) according to the manufacturer’s directions. Sirtuin containing tissue protein samples were tested with the acetylated p53 peptide substrate provided with the kit. Deacetylation of substrate sensitizes it to the color developer releasing the green fluorophore. The enzyme activity was assessed from the fluorescence signal generated in proportion to the amount of deacetylation of the lysine.

### ATP measurement

A bioluminescence assay (ATP determination kit; Invitrogen) kit was used for the quantitative determination of ATP. The assay employed recombinant firefly luciferase and its substrate D-luciferin. Briefly, reaction solution containing luciferase and luciferin was plated and background luminescence measured. ATP standard solution or sample containing ATP was added to respective wells and luminescence was measured. ATP concentration was deduced from the standard curve and normalized to total protein concentration.

### Hexokinase activity

Hexokinase activity was measured using the hexokinase colorimetric assay kit (Sigma-Aldrich. St. Louis, MO) according to the manufacturer’s instructions. Absorbance was measured at 563 nm using a SpectraMax M5 plate reader (Molecular Devices). One unit of hexokinase is the amount of enzyme that will generate 1.0 mM of NADH per min at pH 8.0 and room temperature. The results were normalized to the amount of total protein compared to the sham.

### Western blot analysis

The heart tissues were homogenized in Pierce RIPA lysis buffer (Thermo Scientific, Chicago, IL) containing 25 mmol/L Tris-HCl pH 7.6, 150 mmol/L NaCl, 1% NP-40, 1% sodium deoxycholate, 0.1% SDS and protease inhibitor cocktail (Sigma Aldrich, St. Louis, MO, USA) in a D1000 handheld homogenizer (Benchmark Scientific, Sayreville, NJ, USA). Heart tissue lysates were centrifuged at 14,000 g for 10 min and the supernatant saved for protein estimation and analysis. Protein aliquots were combined with 4X Laemmli buffer (Bio-Rad, Hercules, CA, USA) and resolved on a 10% SDS polyacrylamide gel, transferred to PVDF membrane, blocked using 5% (w/v) nonfat dried milk or 5% BSA in Tris-buffered saline containing 25 mmol/L Tris-HCl (pH 7. 4), 0.13 mol/L NaCl, 0.0027 mol/L KCl and 0.1% Tween 20 for 1 h at room temperature (RT) and then incubated with respective antibodies overnight at 4 °C or for 1 h at RT. The membranes were probed with antibodies to Sirt-1, Pgc-1α, Bip, Pdk, P-AMPK, AMPK, P-ACC, and ACC. The membranes were subsequently washed and incubated with horseradish peroxidase conjugated secondary antibody for 1 h at RT and developed using enhanced western lightning plus-ECL (PerkinElmer). Protein bands developed on X-ray films were scanned from the original film and the Western Blot bands are presented in the manuscript without alteration (Supplementary Figures 3 and 4). The bands were quantified using the ImageJ software (Wayne Rasband, NIH, Rockville, MD, USA).

### JC-1 assay for mitochondrial membrane potential

The experiment was performed using Isolated Mitochondria Staining kit from Sigma Chemical Co., St. Louis, MO. Briefly, 5 µg sample of mitochondria isolated from the heart (Biovision, CA) was stained with a 0.2 µg/mL JC-1 solution. For a control, valinomycin, an ionophore, was added to an identical 5 µg sample of isolated mitochondria to a final concentration of 0.5 μg/ml. This sample was placed on ice for 10 minutes to allow complete dissipation of the membrane potential, and then stained with a 0.2 µg/mL JC-1 solution and assayed in parallel. Samples were read over a period of 30 minutes using a BIOTEK (Synergy HT) fluorescence plate reader (excitation wavelength = 485 nm, emission wavelength = 590 nm). Results are expressed as a percentage of the valinomycin baseline control fluorescence.

### Vascular function assessment

Aorta was rapidly excised and placed in chilled Krebs solution of the following composition (mM): NaCl, 118; NaHCO_3_, 25; glucose, 5.6; KCl, 4.7; KH_2_PO_4_, 1.2; MgSO_4_ 7H_2_O, 1.17 and CaCl_2_ 2H_2_O, 2.5. After periadventitial adhering fat was removed, endothelial intact aorta were cleaned and cut into 2 mm rings. The aortic rings were mounted under resting tension of 10 mN in myograph organ bath chambers (Danish Myo Technology A/S) filled with Krebs solution at 37 °C (pH 7.4) and continuously bubbled with a mixture of 95% O_2_ and 5% CO_2_. Isometric force was recorded using a PowerLab/8SP data acquisition system (Software Chart, version 5, AD Instrument, Colorado Springs, CO, USA). The tissues were allowed to equilibrate for 1 h before starting the experiments.

After equilibration, aortic rings were contracted with high KCl solution (80 mM) to verify the viability of the preparations. After washing out KCl, cumulative concentration-response curves to acetylcholine (ACh; 0.001–10 µM), an endothelium-dependent vasodilator or sodium nitroprusside (SNP; 0.0001–10 µM), a NO donor were generated with aortic rings. The rings were contracted with phenylephrine (PE, an α1-adrenergic receptor agonist). Cumulative concentration-response curve to PE were also performed in aortic rings (0.001–10 µM) from Sham, HI and HI+ DCA treated rats.

### Statistics

Survival analysis was performed by Kaplan-Meier method and significance between survival curves was determined using GraphPad software (La Jolla, CA). Multigroup comparisons were carried out and significance determined by one-way ANOVA followed by Tukey’s test using GraphPad software (Graphpad Prism, WA). Two-group comparisons for significance were done by unpaired t test using GraphPad software. A p value of less than 0.05 is considered significant.

## Electronic supplementary material

Supplementary information
