# Supplementary material for: Mitochondrial targeting by dichloroacetate improves outcome following hemorrhagic shock
Source: Sci Rep. 2017 Jun 1;7:2671. doi: 10.1038/s41598-017-02495-5 (PMC5453974; doi:10.1038/s41598-017-02495-5)
Supplement: Supplementary file 1 — Supplementary information [file 41598_2017_2495_MOESM1_ESM.doc]

Mitochondrial targeting improves outcome following hemorrhagic shock

Kumar Subramani^1^, Sumin Lu^1^, Marie Warren^1^, Xiaogang Chu^1^, Haroldo A. Toque^4^, R. William Caldwell^4^, Michael P. Diamond^5^ and [Raghavan Raju](http://www.ncbi.nlm.nih.gov/pubmed/?term=Raju R%5Bauth%5D)^1,2,3,*^

^1^Departments of Laboratory Sciences, ^2^Surgery, ^3^Biochemistry and Molecular Biology, ^4^Pharmacology and Toxicology, ^5^Department of Obstetrics and Gynaecology, Augusta University, Augusta, GA 30912, United States of America.

^*^Corresponding author:

Raghavan Raju, Ph.D.

Augusta University,
CB 2601, Sanders Building
1479 Laney Walker Blvd.
Augusta, GA – 30912
Phone: 706-723-4138
Fax: 706-721-8293

**Supplementary Figures**

**A**


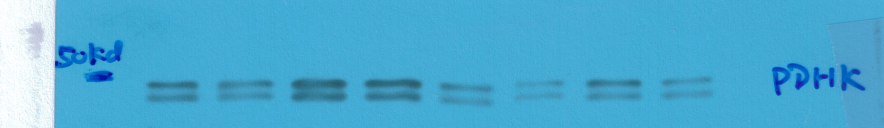


50


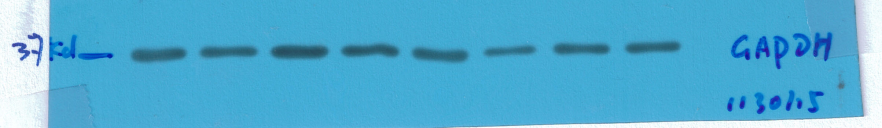


37

GAPDH

PDK

HI + Veh

Sham

HI + DCA

10mg

25mg

**Supplementary Figure 3.** Original western blot films to the corresponding figures. Molecular weight markers are indicated in kDa.


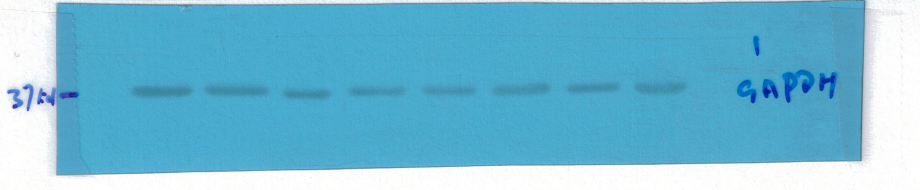

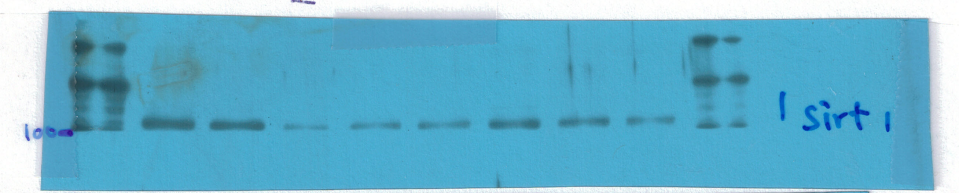


**A**

GAPDH

Sirt-1

100

37

150

HI + Veh

Sham

HI + DCA

10mg

25mg

**Supplementary Figure 4.** Original western blot films to the corresponding figures.
Molecular weight markers are indicated in kDa.


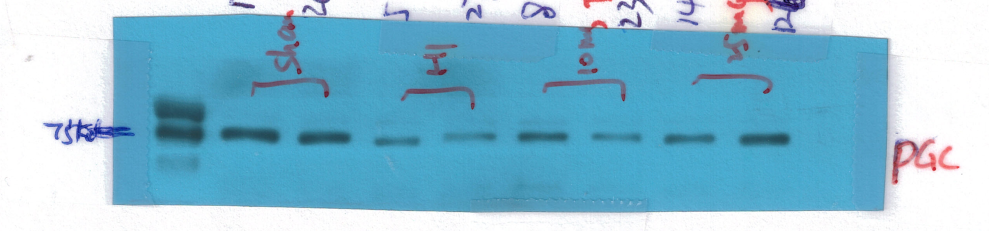

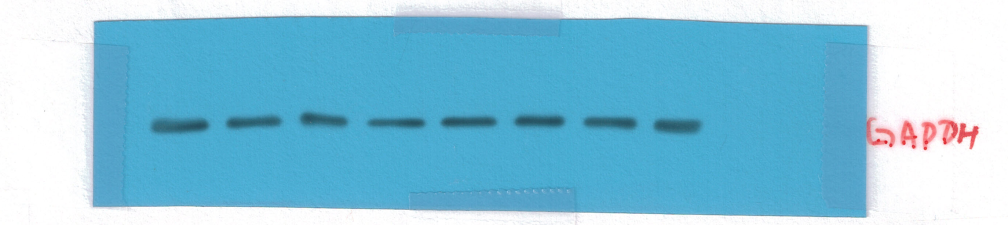


GAPDH

PGC

37

**B**

**Supplementary Figure 4.** Original western blot films to the corresponding figures.
Molecular weight markers are indicated in kDa.
